# Profiling Class-Wide Bioactivities of Flavonoids While Minimizing Compound-Specific Effects Using an Equimolar Mixture Strategy

**DOI:** 10.1021/acs.jafc.5c09562

**Published:** 2025-10-23

**Authors:** Alex Xiong Gao, Amy Xiao-Yang Wang, Zheng-Qi Wang, Mei-Xia Yang, Man-Man Sun, Farkhod Eshboev, Jin Gao, Tina Ting-Xia Dong, Karl Wah-Keung Tsim

**Affiliations:** † Division of Life Science, 611834 The Hong Kong University of Science and Technology, Clear Water Bay, Hong Kong 999077, China; ‡ Shenzhen Key Laboratory of Edible and Medicinal Bioresources, HKUST Shenzhen Research Institute, Hi-Tech Park, Nanshan, Shenzhen 518000, China; § Key Laboratory of High Magnetic Field and Ion Beam Physical Biology, 53040 Hefei Institutes of Physical Science, Chinese Academy of Sciences, Hefei 230031, China

**Keywords:** flavonoids, polyphenols, phytonutrients, cocktail, class-effect, neurite outgrowth

## Abstract

Flavonoids share a C_6_–C_3_–C_6_ core yet vary in side-chain decorations. Here, we tested whether an equimolar cross-subclass mixture could serve as a “class reference” by capturing class-wide bioactivities and diluting outlier effects. Twenty flavonoids across five subclasses were blended and tested alongside five single-flavonoid representatives—luteolin, quercetin, naringenin, EGCG, and genistein. In rat PC12 pheochromocytoma cells, the flavonoid mixture enhanced NGF-induced neuronal differentiation and activated reporters of neurofilament, cAMP, NF-κB, and antioxidant response elements, paralleling the effects of the representatives and indicating conserved neurotrophic activity. All flavonoidic samples were nontoxic at 5 μM but became toxic at 50 μM, except naringenin. Notably, luteolin-induced mitochondrial depolarization and cytotoxicity were buffered as the proportion of other flavonoids increased. The mixture bound amyloid-β_1–42_ ∼10× more weakly than EGCG, evidencing dilution of a specific interaction. This proof-of-concept study offers tools and a framework to map flavonoids’ functions in nutritional and pharmacological contexts.

## Graphical abstract

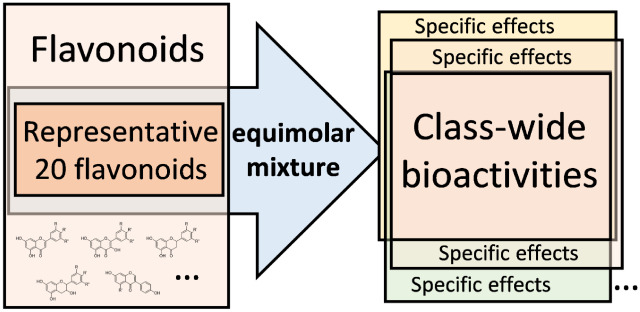

## 1 Introduction

Flavonoids are ubiquitous plant secondary metabolites that enter the human diet primarily through fruits, vegetables, and medicinal herbs.^1^ To date, over 10,000 flavonoid molecules have been identified, most of which can be classified into several major subclasses: i.e., flavonols, flavones, flavanols, flavanones, and isoflavones, characterized by variations in the oxidation patterns and functional group decorations on a conserved C_6_–C_3_–C_6_ scaffold.^2^ The consumption of flavonoids has been reported to have numerous health benefits, including reduced risks of inflammatory, neurodegenerative, and cardiovascular diseases, as well as certain cancers.^3^ Luteolin, quercetin, epigallocatechin gallate (EGCG), genistein, and numerous other flavonoids have garnered significant attention in nutritional and pharmacological research since the 1990s.^4^

Neurotrophic function refers to neurotrophin-driven effects that support neuronal growth, differentiation, survival, and maintenance. Several lines of evidence link the neuroprotective and neuromodulatory benefits of flavonoids to neurotrophin-like actions. Despite typically low oral bioavailability, a number of flavonoid aglycones exhibit measurable blood–brain barrier permeability, enabling possible direct brain actions.^5^ Consistent with this possibility, *in vitro* and *in vivo* models have shown that flavonoids can engage pathways implicated in neuronal plasticity and survival, including MEK–ERK and PI3K-AKT signaling cascades, and under specific conditions, which upregulate expressions of neurotrophins, such as BDNF, and its downstream TrkB signaling.^6^

The conserved C_6_–C_3_–C_6_ scaffold implies that certain molecular interactions and signaling events may be shared across the flavonoid family, as evidenced by numerous studies highlighting their widely recognized antioxidative and anti-inflammatory properties.^7^ However, the variations in ring oxidation, glycosylation, and other side-chain modifications introduce physicochemical diversity, potentially conferring unique bioactivities to individual compounds,^8^ which cannot be extrapolated to the entire class. Despite this, current research has disproportionately focused on individual flavonoids, particularly in studies exploring their mechanisms of action in specific disease contexts. This focus has generated a wealth of data but also poses challenges in identifying genuine class-wide bioactivities in distinguishing them from compound-specific effects.

To address this limitation from a systematic perspective, we proposed an equimolar “class reference” mixture as a proof-of-concept verification. The mixture comprised 20 flavonoids, with four representatives from each major subclass. In the mixture, the concentration of each individual flavonoid was reduced to one-twentieth of the flavonoid concentration. The mixture of 20 flavonoids was then benchmarked against five representative single compounds in cultured rat PC12 pheochromocytoma cells. We hypothesized that the class-wide bioactivities of flavonoids would remain as robust in the mixture as in the single flavonoids, whereas less conserved effects would be attenuated. Neurotrophic effects, cytotoxicity, mitochondrial membrane potential (MMP), and amyloid-β_1–42_ (Aβ_1–42_) binding were evaluated to validate the functional concordance of the mixture. This study introduces a practical tool in distinguishing genuine class-wide flavonoid bioactivities from compound-specific variations, offering a novel framework to advance both mechanistic studies and translational applications in phytonutrient research.

## 2 Methods

### 2.1 Chemicals

All 20 flavonoids (>98% purity, determined by HPLC) were obtained from Chengdu DeSiTe Biological Technology (Chengdu, China). Each compound was dissolved in DMSO at 50 mM and stored at −20 °C. Equal volumes of the 20 stocks were mixed to produce a 20-flavonoid mixture in which the total flavonoid concentration remained 50 mM, and each compound was diluted 20-fold to 2.5 mM. Propidium iodide (PI), carbonyl cyanide 4-(trifluoromethoxy)­phenylhydrazone (FCCP), tetramethylrhodamine ethyl ester perchlorate (TMRE), and Hoechst 33342 were obtained from MedChemExpress (Monmouth Junction, NJ). JC-1 was obtained from Yuanye Biotechnology (Shanghai, China). 3-(4,5-dimethylthiazol-2-yl)-2,5-diphenyltetrazolium bromide (MTT) was purchased from Sigma-Aldrich (St. Louis, MO). Native mouse NGF 2.5S protein was purchased from Alomone Laboratories (Jerusalem, Israel). All cell culture reagents were sourced from Thermo Fisher Scientific (Waltham, MA). Biotinylated Aβ_1–42_ was purchased from GenScript (Nanjing, China).

### 2.2 Cell Cultures

PC12 cell cultures were maintained in a humidified incubator at 37 °C with 5% w/v CO_2_. The cells were cultured in Dulbecco’s Modified Eagle Medium (DMEM) supplemented with 6% fetal bovine serum (FBS), 6% horse serum (HS), 100 U/mL penicillin, and 100 μg/mL streptomycin.^9^ The cells were cultured in 100 mm culture dishes and passaged by trypsinization every 2–3 days. For all experiments, except for neuronal differentiation, the culture plates were precoated with 50 μg/mL poly-l-lysine to enhance cell attachment and to promote uniform cell distribution.

### 2.3 Transfection of DNA Constructs and Luciferase Assay

A jetPRIME (Polyplus, New York, NY) reagent was used for the transfections of pNF68-Luc, pCRE-Luc, pARE-Luc, and pNF-κB-Luc constructs into PC12 cell cultures. After 12 h of transfection, the medium was replaced with DMEM (supplemented with 1% FBS and 1% horse serum) containing the indicated drugs for 24 h of treatment. A luciferase assay was performed using a commercial kit (Promega, Madison, WI), and the luminescence intensity was measured using a luminometer (Promega).

### 2.4 Neuronal Differentiation

PC12 cells (2 × 10^4^ cells/mL) were seeded onto a 6-well plate and cultured for 24 h. The medium was then replaced with a low-serum medium (1% HS and 1% FBS) for an additional 24 h of cultivation prior to flavonoid and NGF treatments. Two days later, images were acquired using a phase-contrast microscope (Carl Zeiss, Oberkochen, Germany). The proportion of neurite-bearing cells in each well was analyzed based on at least five randomly selected fields of view. A cell was considered differentiated with a neurite that was longer than the diameter of its cell body.

### 2.5 MTT Assay and PI Staining

For the measurement of cell viability, cells were seeded in 96-well plates at a density of 1 × 10^5^ cells/mL and cultured for 24 h. The medium was then replaced with basal DMEM containing the respective drug treatment, followed by incubation for 48 h. MTT was prepared in basal DMEM at a final concentration of 0.5 mg/mL and added to the cells by replacement of the culture medium. After 4 h of incubation, DMSO was added to dissolve the formazan crystals, and the absorbance at 570 nm was measured using a microplate reader. Absorbance values were adjusted by subtracting the blank well readings and normalizing them to the untreated control group. To measure the proportion of dead cells with PI staining, the culture medium was replaced with HBSS containing Hoechst 33342 (5 μg/mL) and PI (2 μg/mL) after 48 h of drug treatment. Cells were stained for 15 min, and the images were captured using a Celldiscoverer 7 imaging system (Carl Zeiss) with a 0.5× objective and a 2× tube lens. The ratio of PI-positive cells to total nuclei was calculated for each field to determine the proportion of dead cells.

### 2.6 Determination of MMP Depolarization

The probes, JC-1 and TMRE, were used to determine acute MMP alterations. The JC-1 probe (1 μg/mL) was loaded into the cell culture with HBSS for 20 min. The cells were then washed and incubated with the indicated drugs diluted in HBSS. After 20 min of incubation at 37 °C, the fluorescence intensity ratio of red (Ex: 525 nm; Em: 595 nm) to green (Ex: 490 nm; Em: 530 nm) was measured using a FlexStation 3 (Molecular Devices, San Jose, CA). For TMRE staining, the culture medium was replaced with HBSS containing 20 nM TMRE. After incubation for 20 min, the drugs were directly added to the culture for 20 min. Fluorescence images were acquired (Nikon, Tokyo, Japan) using a 20× objective in the slow scan mode.

### 2.7 Biolayer Interferometry (BLI) Analysis

The interaction between the EGCG/flavonoids mixture and A_β1–42_ protein was analyzed using the Octet R8 system (Sartorius, Göttingen, Germany). Biotinylated Aβ_β1–42_ was immobilized onto streptavidin biosensors following an experimental procedure consisting of a baseline (60 s), load (460 s), and baseline 2 (60 s). PBS with 0.02% Tween-20 (pH 7.4) was used as the running buffer. EGCG and the flavonoids mixture, prepared as 50 mM stock solutions in DMSO and diluted to appropriate concentrations, were tested for binding through the steps of baseline (60 s), association (60 s), and dissociation (60 s). Data were analyzed using the double reference subtraction method to determine binding kinetics.

### 2.8 Statistics

Statistical analyses were performed by using GraphPad Prism software. To compare multiple groups, one-way ANOVA followed by Dunnett’s post hoc test was used. Prior to conducting ANOVA, the Shapiro–Wilk test was applied to assess normality, and the Brown–Forsythe test was used to evaluate homoscedasticity. If the data did not meet the normality assumption, the Kruskal–Wallis test was applied as a nonparametric alternative. In cases where the assumption of equal standard deviations (SDs) was violated, Welch’s ANOVA was employed. Results are presented as mean ± SD. Statistical significance was determined as (∗) when *p* < 0.05.

## 3 Results

### 3.1 Mixture Strategy Reflects the Neurotrophic Effects of Representative Flavonoids

Based on current research interests, we selected five of the most commonly investigated flavonoid subclasses: flavones, flavonols, flavanones, flavanols, and isoflavones (Figure 1). Each subclass includes four widely investigated compounds; most of which are commonly found in food sources and largely conform to their original aglycone backbones. For each subclass, we chose a representative flavonoid based on the highest number of PubMed citations: luteolin (flavone),^10^ quercetin (flavonol),^11^ naringenin (flavanone),^12^ EGCG (flavanol),^13^ and genistein (isoflavone).^14^ As of June 2025, each of these has been cited in at least 4500 articles. The equimolar mixture of 20 flavonoids was abbreviated as MIX_20_ (Figure 1). The five representative flavonoids were mixed as MIX_5_, and the remaining 15 flavonoids were designated as MIX_15_.

**Figure 1:**
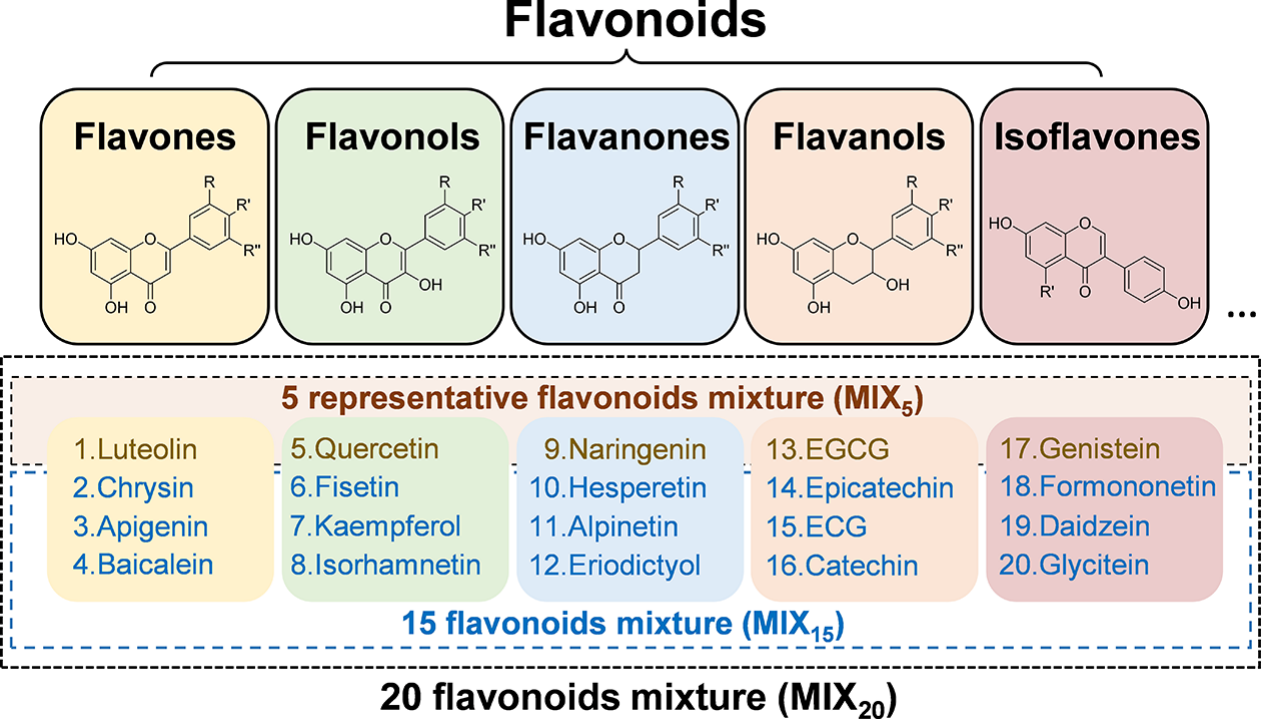
Preparation of equimolar flavonoid mixtures across the five subclasses. Flavonoids from five major subclasses—flavonols, flavones, flavanols, flavanones, and isoflavones—were used to formulate an equimolar 20-flavonoid mixture, i.e., MIX_20_. Five key flavonoids (quercetin, luteolin, naringenin, EGCG, and genistein) were selected to create a five-representative flavonoid mixture, i.e., MIX_5_, while the remaining 15 flavonoids were used to generate the 15-flavonoid mixture, i.e., MIX_15_.

Many flavonoids are reported to possess neurotrophic effects; thus, PC12 cells were employed as a model to test the activities.^6^ First, we employed luciferase reporters of neurofilament-68, cAMP response element (CRE), antioxidant response element (ARE), and NF-κB as indicators of neurotrophic activities.^15^ The flavonoid mixture having 20 flavonoids (MIX_20_) dose-dependently activated the expression of these promoters, increasing their activities by 2- to 4-fold (Figure 2A). The MIX_5_, the positive control NGF at 50 ng/mL, and the individual representative flavonoids—luteolin, quercetin, naringenin, and genistein (each at 10 μM)—also significantly increased the promoter activities (Figure 2B).

**Figure 2:**
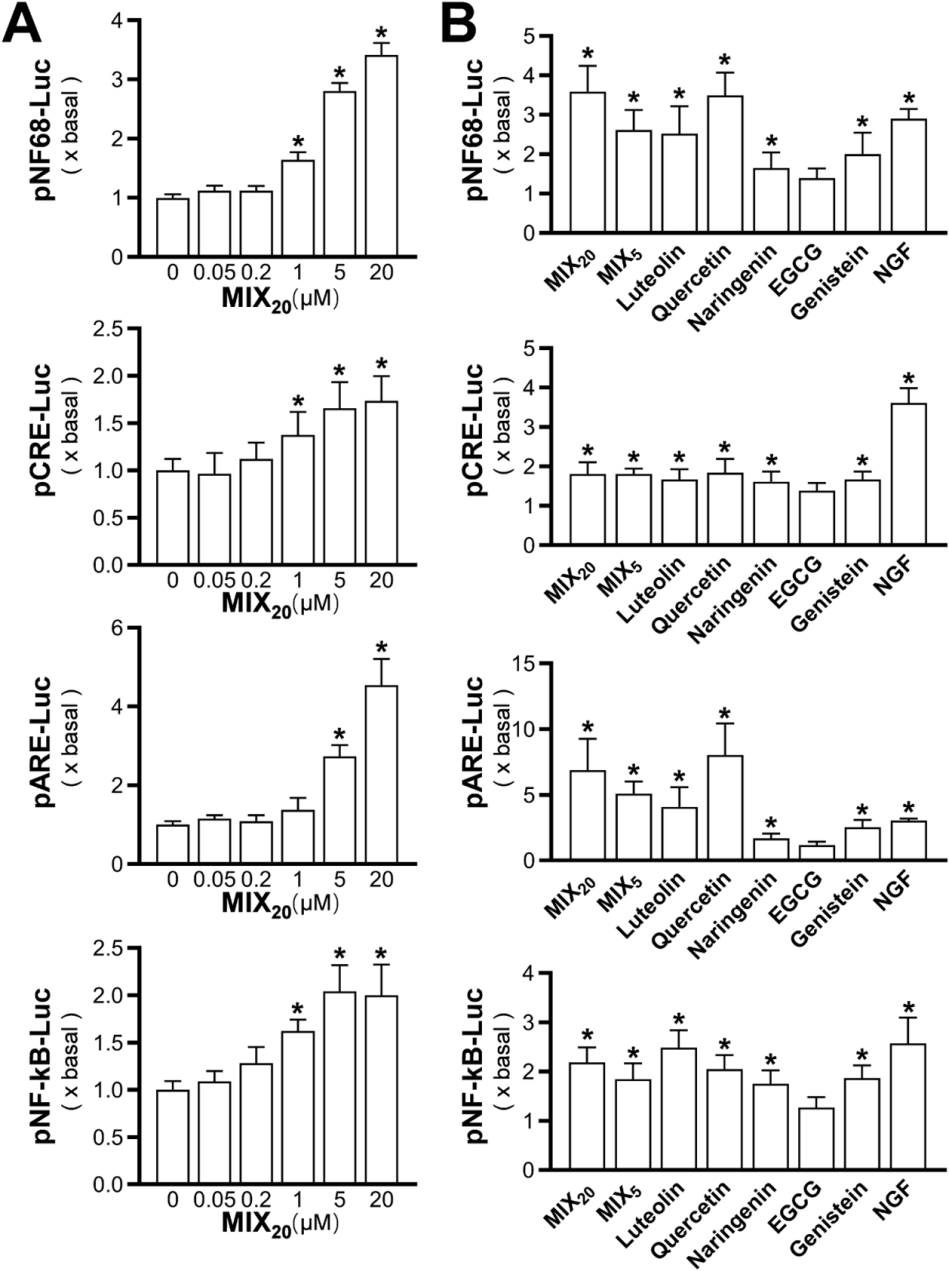
Activation of neurotrophic promoters by the flavonoid mixture in cultured PC12 cells. PC12 cells were transfected with luciferase constructs for neurofilament-68 (pNF68-Luc), the cAMP response element (pCRE-Luc), the antioxidant response element (pARE-Luc), and NF-κB (pNF-κB-Luc). After transfection, cells were treated with (A) different doses of the mixture or (B) 10 μM of individual representative flavonoids, as indicated, for 24 h in DMEM containing 1% FBS and 1% horse serum. Cells were then lysed, and luciferase activity was measured by a luminometer. NGF (50 ng/mL) was a control. Values are expressed as fold change and presented as mean ± SD (*n* = 3–8). * *p* < 0.05 compared to control.

Many flavonoids have been reported to potentiate the NGF-induced neurite outgrowth in PC12 cells, a key indicator of neurotrophic properties.^16,17^ Here, MIX_20_ dose-dependently increased the ratio of differentiated cells when coapplied with 1.5 ng/mL NGF (Figure 3A,B). NGF at 50 ng/mL, used as a positive control, led to differentiation in approximately 70% of cells. Consistent with previous findings, 5 μM luteolin combined with 1.5 ng/mL NGF showed a strong effect, inducing neuronal differentiation of PC12 cells to approximately 35% (Figure 3A,C).^16,18^ MIX_20_, quercetin, and genistein also significantly induced differentiation, reaching about 20%. These results demonstrate that MIX_20_ can effectively reflect the neurotrophic properties of flavonoids.

**Figure 3:**
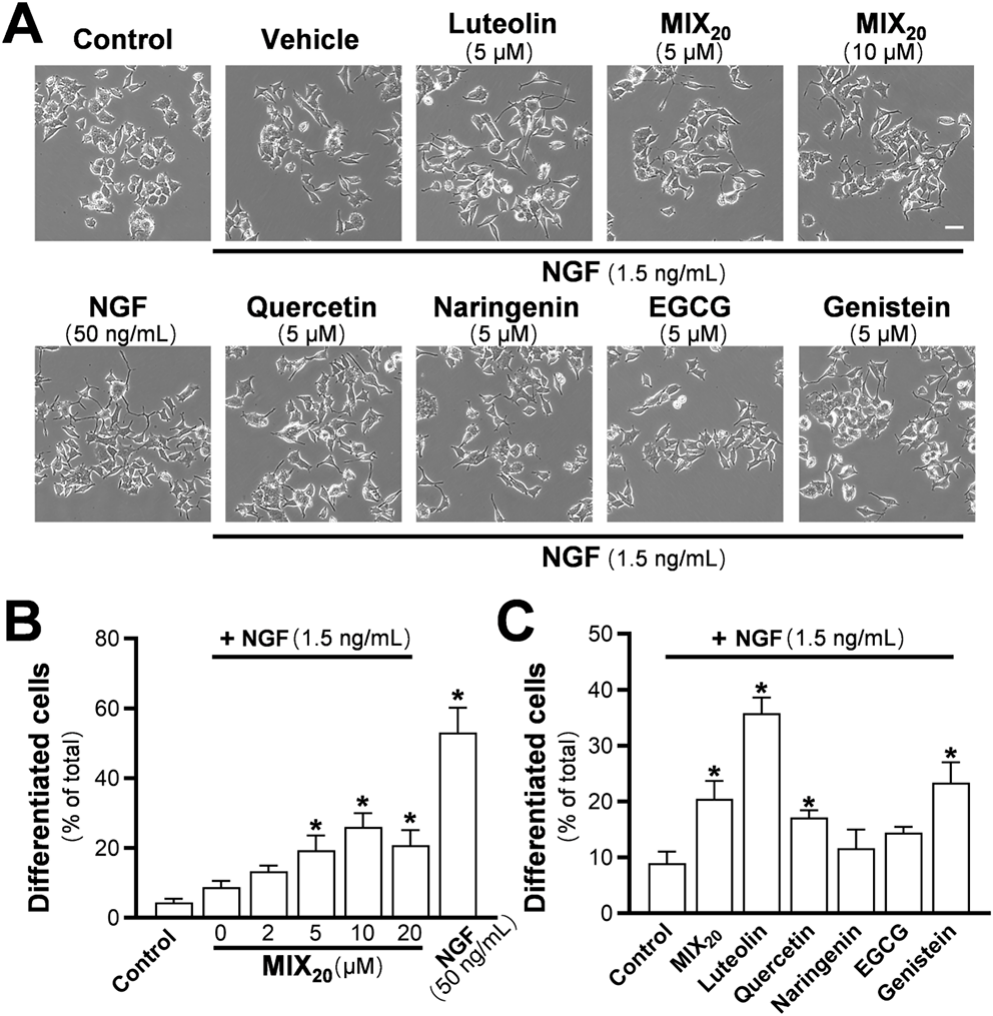
Flavonoid mixture potentiates NGF-induced neuronal differentiation in PC12 cells. PC12 cells were seeded and cultured for 24 h. The medium was then replaced with low-serum DMEM (1% FBS and 1% horse serum) for an additional 24 h prior to treatment with flavonoids in the presence of 1.5 ng/mL NGF. After 2 days of treatment, images were captured, and (A) representative images of differentiated cells are shown. The proportion of neurite-bearing cells was analyzed with neuronal differentiation defined as cells possessing neurites longer than the diameter of the cell body. The quantification results for the (B) dose–response curve of the MIX_20_ and (C) comparisons among different representative flavonoids at 5 μM are shown. NGF (50 ng/mL) was used as a control. Scale bar: 50 μm. Values are expressed as percentage of control and presented as mean ± SD (*n* = 3). * *p* < 0.05 compared to control.

### 3.2 Mixture Strategy Reflects the Toxicity of Representative Flavonoids

Many flavonoids are known to exhibit cytotoxicity at high concentrations, although this insight has been particularly emphasized in cancer research.^19^ Therefore, it is important to assess whether the mixture exhibits toxicity similar to that of other representative flavonoids. After 48 h of culture in basal nonserum DMEM, MIX_20_ showed compromised cell viability at 50 μM, with a 25% decrease (Figure 4A). At 5 μM, none of the representative flavonoids exhibited toxicity (Figure 4B). By contrast, except for naringenin, all representative flavonoids significantly inhibited cell viability at 50 μM, indicating the toxicity pattern of flavonoids. The toxicity of the mixture was further evaluated using PI staining, which revealed that MIX_20_ induced a higher percentage of dead cells at 10 μM compared to the control (Figure 4C), although the overall cell viability measured by MTT at this concentration remained unchanged. The percentage of dead cells increased further at 20 μM and 50 μM.

**Figure 4:**
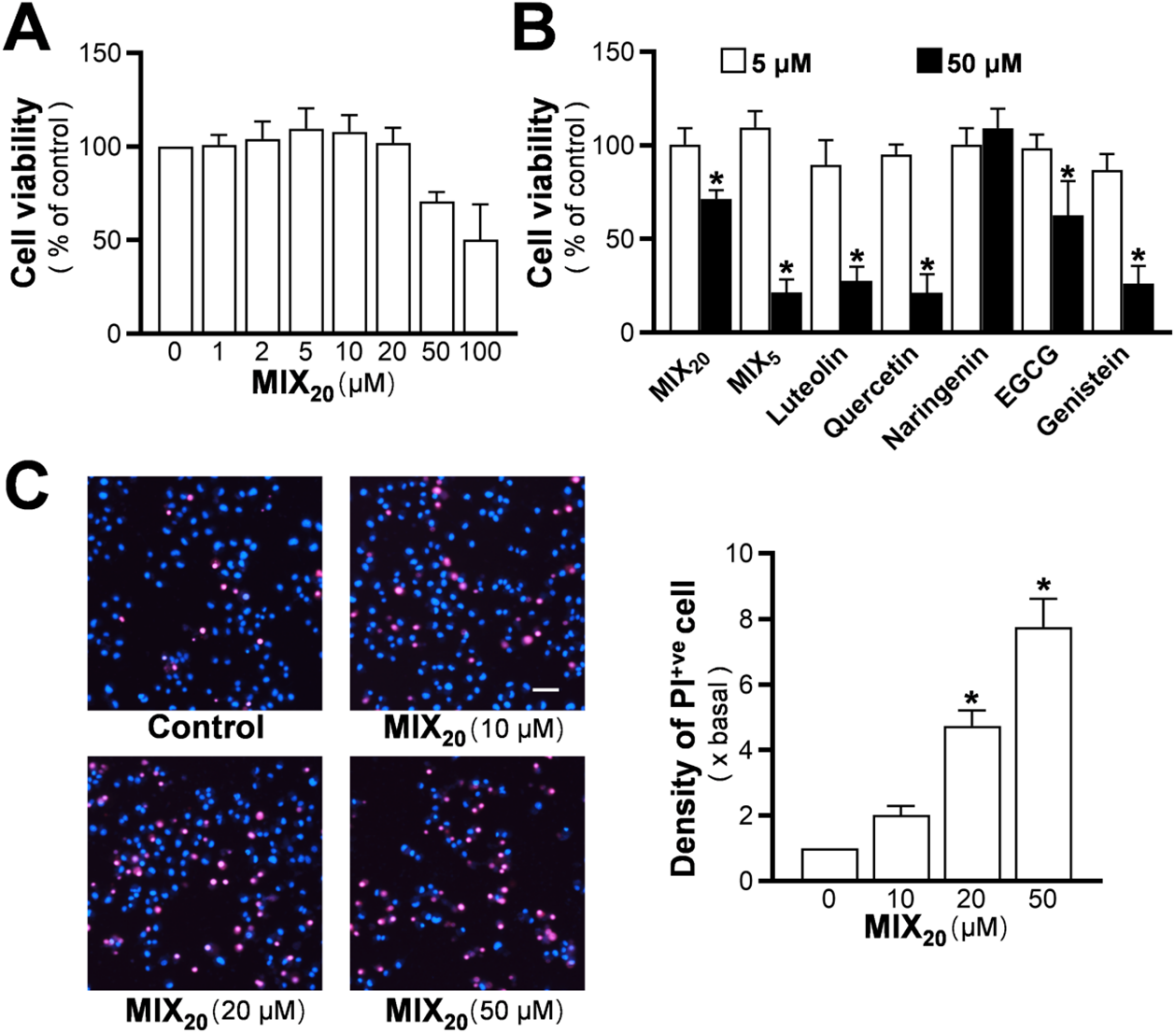
Evaluation of the toxic properties of the flavonoid mixture in PC12 cells. (A) PC12 cells were treated with MIX_20_ at different doses in DMEM for 48 h, and the MTT assay was performed to assess cell viability. (B) The effects of the MIX_20_, MIX_5_, and representative flavonoids on cell viability in PC12 cells were compared at concentrations of 5 and 50 μM. (C) PC12 cells were treated with MIX_20_ for 48 h under serum-free conditions, and PI/Hoechst 33342 staining was used to determine the percentage of cell death. Representative images are shown. Scale bar = 50 μm. Values are expressed as percentage of control or fold change and presented as mean ± SD (*n* = 3–8). * *p* < 0.05 compared to control.

Based on the neurotrophic and toxic properties shared among the mixture and representative flavonoids, we next sought to determine whether the flavonoid-induced effects of the mixture could reach a saturation state, where further enhancement may not be possible by adding additional flavonoids. This notion was tested in MIX_15_ that did not have luteolin, quercetin, naringenin, EGCG, and genistein. In the dose-dependent study, 20 μM MIX_15_ exhibited almost saturated activation of the pNF68-Luc promoter (Figure 5A). However, when 5 μM of the representative flavonoids (luteolin, or quercetin, or naringenin, or EGCG or genistein) were added to the 20 μM MIX_15_, attaining a total flavonoid concentration of 25 μM, the NF68 promoter activity was further increased by quercetin, naringenin, and EGCG, but not by luteolin or genistein (Figure 5B).The addition of NGF achieved the highest enhancement of the readout, indicating that the luciferase system itself was not a limiting factor. On the other hand, no cell toxicity was observed at concentrations lower than 50 μM of MIX_15_. At 50 μM, MIX_15_ began to cause a minor decrease in MTT assay readings, suggesting that it was a threshold concentration (Figure 5C). Adding 5 μM individual representative flavonoids to reach a total flavonoid concentration of 55 μM resulted in enhanced toxicity, except in the case of naringenin (Figure 5D). These results suggest that the mixture, at a relatively functional saturation concentration, leads to a convergence of flavonoid-induced functions. However, the readout remains complex due to potential interactions among the molecules and crosstalk between cell signaling pathways.

**Figure 5:**
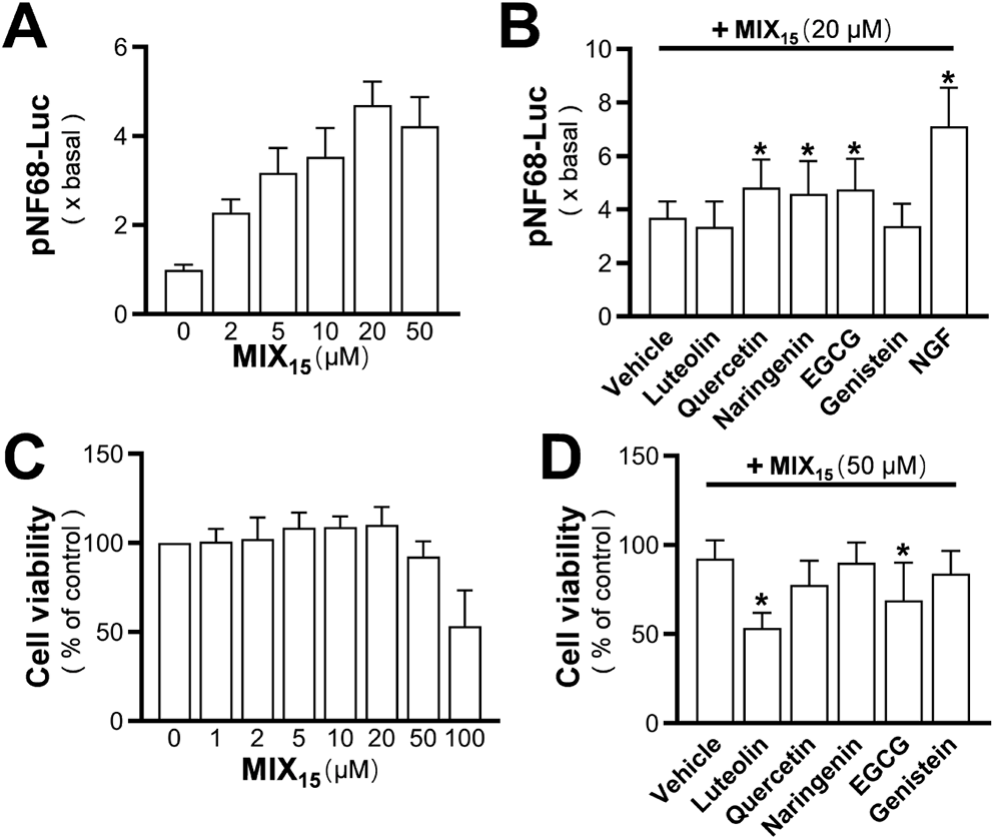
Effect of threshold concentrations of MIX_15_ with representative flavonoids on cell viability and promoter activation. (A) PC12 cells were transfected with the pNF68-Luc construct, followed by treatment with MIX_15_ at different concentrations for 24 h to induce NF68 promoter activation. Luciferase activity was measured. (B) NF68 promoter activation was analyzed upon treatment with MIX_15_ alone or in combination with 5 μM representative flavonoids. (C) The cell viability of Mix-R-treated cells was evaluated by the MTT assay after 48 h of treatment in basal DMEM. (D) PC12 cells were treated with MIX_15_ at 50 μM combined with 5 μM representative flavonoid for 48 h. Values are expressed as percentage of control or fold change and presented as mean ± SD (*n* = 3–8). * *p* < 0.05 compared to group of MIX_15_ treatment.

### 3.3 Mixture Strategy for Diluting and Validating the Specificity of Flavonoid Functions

As flavone and flavonol have been shown to instantly decrease the level of MMP, we then assessed whether MIX_20_ could exhibit this effect. Indeed, MIX_20_ dose-dependently induced a reduction in MMP (Figure 6A).^20^ As expected, luteolin and quercetin significantly decreased MMP, whereas the other three representative flavonoids showed no significant impact, consistent with our previous study (Figure 6B).^20^ These results were further validated using the TMRE method, with FCCP serving as a positive control to fully diminish the MMP (Figure 6C).

**Figure 6:**
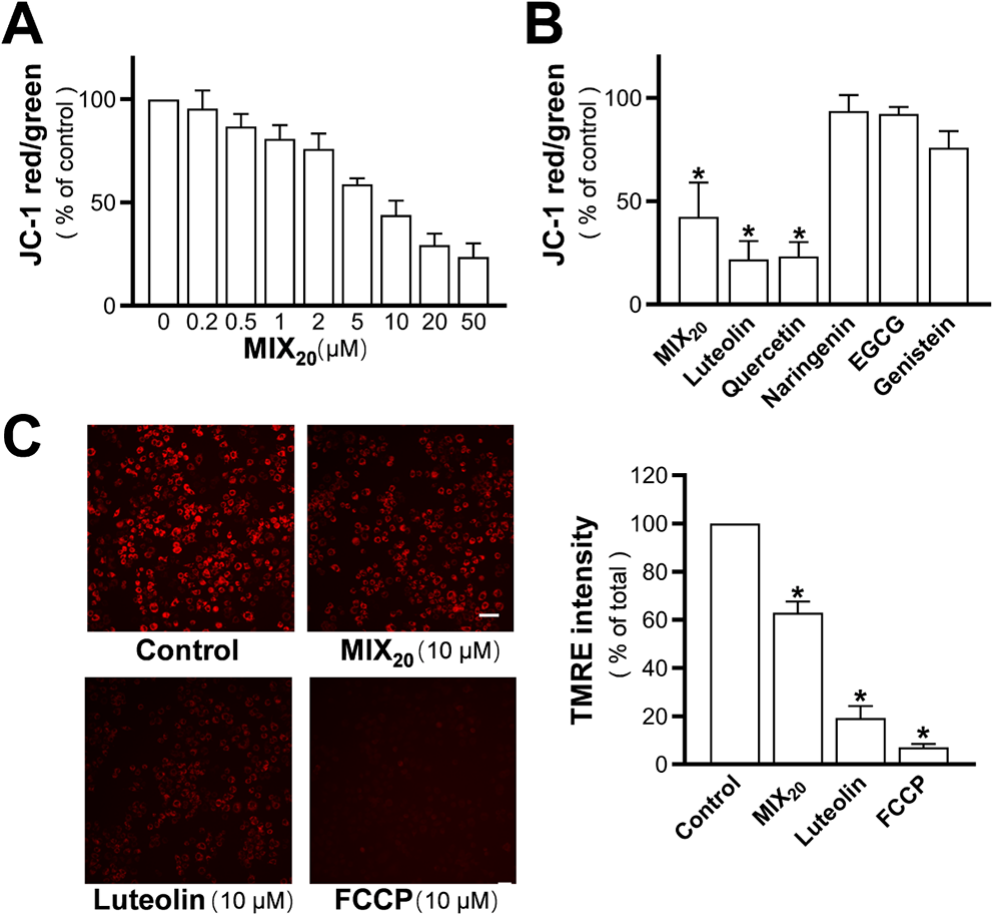
Effects of the flavonoid mixture on the mitochondrial membrane potential (MMP) in PC12 cells. (A) PC12 cells were loaded with JC-1 dye (1 μg/mL) and treated with MIX_20_ in HBSS for 20 min. The red-to-green fluorescence ratio, representing MMP, was measured by using a flexstation microplate reader. (B) MMP changes induced by MIX_20_ and representative flavonoids were compared at 10 μM. (C) PC12 cells were loaded with TMRE (20 nM) and then treated with MIX_20_ or luteolin at 10 μM for 20 min. Fluorescence images were captured (left), and quantitative analysis of the MMP changes was performed (right). FCCP at 10 μM was used as a positive control for the MMP disruption. Scale bar = 50 μm. Values are expressed as percentage of control and presented as mean ± SD (*n* = 4–7). * *p* < 0.05 compared to control.

In terms of induced MMP loss, a possible scenario could be that less functional compounds in the mixture led to a weaker overall effect of the mixture, i.e., a reduced amount of luteolin. To verify this potential dilution effect, luteolin, as a flagship molecule in inducing MMP loss, was directly combined with single flavonoids. When luteolin was paired with naringenin, EGCG, and genistein in a 1:1 ratio, its effect was significantly diluted, possibly because these flavonoids did not induce MMP loss (Figure 7A). Similarly, the addition of luteolin to MIX_20_ slightly reduced luteolin’s effect on MMP. This dilution effect was further tested with cell viability, where luteolin showed significantly reduced toxicity when combined with other flavonoids or the mixture, further supporting the hypothesis (Figure 7B).

**Figure 7:**
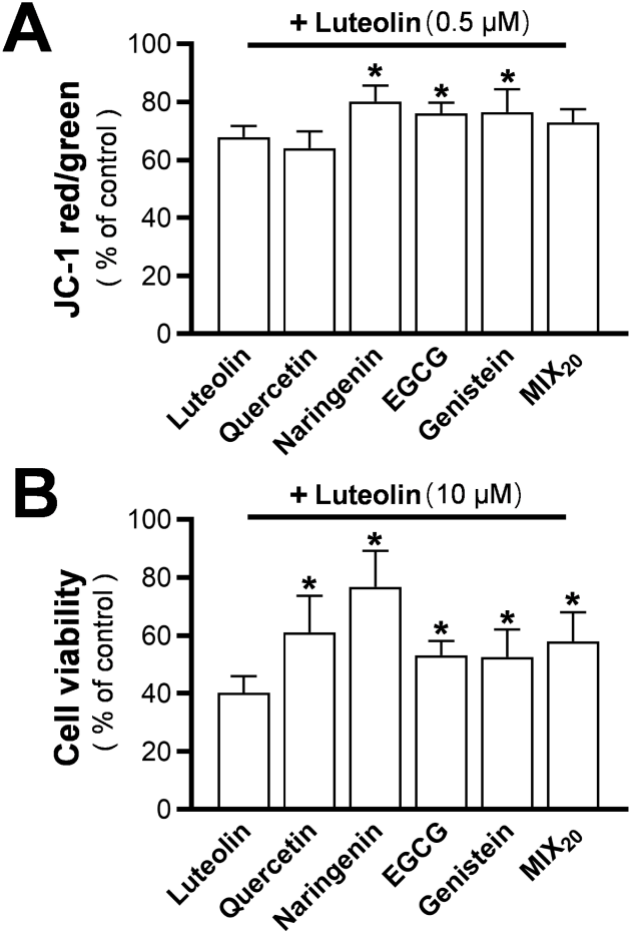
Attenuation of luteolin-induced MMP loss and toxicity by other representative flavonoids. (A) PC12 cells were loaded with JC-1 dye (1 μg/mL) and treated with luteolin alone or in a 1:1 combination with other representative flavonoids in HBSS for 20 min. The total flavonoid concentration was 1 μM. The red-to-green fluorescence ratio was measured by using a FlexStation microplate reader. (B) After 48 h of treatment with luteolin and representative flavonoids at a total concentration of 20 μM in basal DMEM, cell viability was analyzed using the MTT assay. Values are expressed as a percentage of control and presented as mean ± SD (*n* = 8). * *p* < 0.05 compared to group of luteolin treatment.

Based on the above tests, the composition of the mixture appears to dilute the less conserved functions exhibited by certain flavonoids. Therefore, we sought to use the mixture as a reference to investigate a function that has not been widely tested for most flavonoids. EGCG has the most substantial evidence supporting its potential interaction with Aβ.^21^ While many flavonoids have been reported to exhibit anti-Aβ activity, few have been shown to directly bind to Aβ. We employed the BLI system to evaluate the binding affinity of the flavonoid mixture, or EGCG, with Aβ_1–42_ monomers (Figure 8A,B). The results revealed that EGCG indeed has a specific binding affinity for Aβ_1–42_, with a KD of 93.0 μM. By contrast, the mixture demonstrated a weaker affinity, approximately ten times lower than that of EGCG. This weaker interaction may reflect the intrinsic characteristics of the polyphenol structure and hydroxyl groups, which enable broad but weaker interactions with macromolecules.

**Figure 8:**
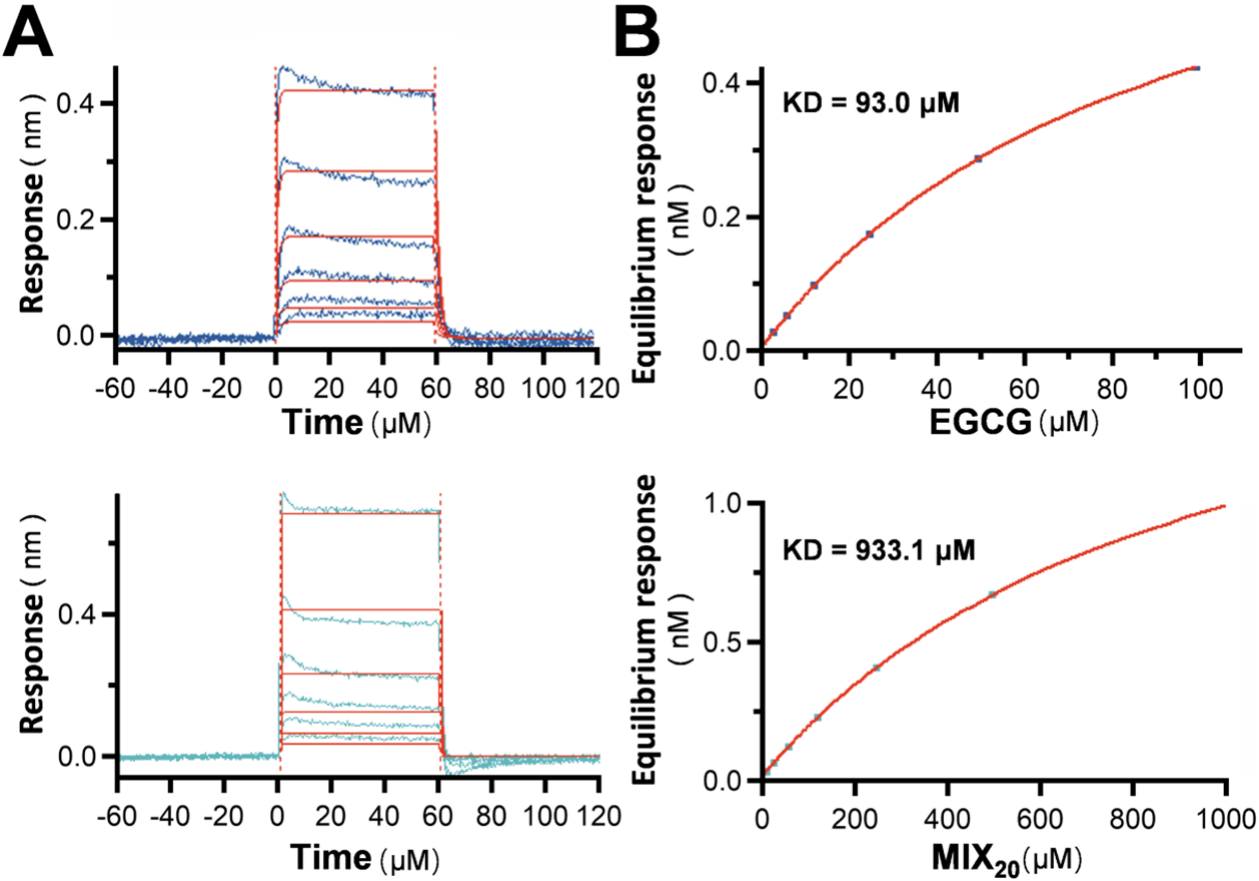
Binding affinities of EGCG and the flavonoid mixture to Aβ_1–42_. The binding affinities of EGCG and the MIX_20_ to Aβ_1–42_ monomers was analyzed using the Bio-Layer Interferometry (BLI) system. (A) A streptavidin biosensor was preloaded with biotinylated Aβ_1–42_, and EGCG or MIX_20_ was then applied to the biosensor. Binding responses were observed in a dose-dependent manner. (B) The steady-state binding data were fitted to a 1:1 binding model to calculate the dissociation constant (KD). EGCG demonstrated a KD of 93.0 μM, indicating a higher binding affinity, whereas MIX_20_ showed a significantly weaker KD of 933.1 μM.

## 4 DISCUSSION

The study aimed to determine whether the health benefits of flavonoids, a family of compounds sharing a common backbone structure, can be understood through a holistic profile. To achieve this, 20 representative flavonoids were selected and pooled together as a “class reference” to evaluate their effects on PC12 cells. Our results confirm that the flavonoid mixture, i.e., MIX_20_, replicates most of the effects observed in the individual flavonoid representatives, particularly in terms of neurotrophic effects and cell toxicity, indicating a strong alignment between the holistic mixture and its individual components. The proposed mixture-based research strategy, along with the functional collective entity demonstrated in this study, could promote more comprehensive functional and mechanistic exploration of flavonoids or their specific subpopulations.

This mixture approach is reasonable as it is supported by the following assumptions and accepted principles: (i) similar chemical structures correspond to similar targets; (ii) flavonoids generally have biological benefits; and (iii) flavonoids tend to exert effects through multiple signaling pathways.^22^ Consequently, by combining different flavonoids, their benefits may overlap or accumulate through shared signaling pathways, whereas specific effects may be weaker or masked, acting as noise. The application of equimolar proportions and even distribution of flavonoids across five subclasses was designed to minimize the bias, i.e., effectively buffering the less conserved functional effects, such as MMP loss and Aβ binding, as being identified here.

This unique approach differs from conventional flavonoid research in the fields of nutrition, food science, and pharmacology. Most existing studies focusing on single compounds often frame within a reductionist biomedical research paradigm that emphasizes specific molecular targets or signaling pathways to address particular diseases.^23^ On the other hand, many studies are aiming to compare different flavonoids in identifying those with better bioactivity. While this strategy can elucidate the structure–function relationships,^24^ the vast number of candidates often limits the depth of insights that can be achieved. Additionally, many studies utilize flavonoids or flavonoid-enriched products.^25^ Although such methods offer direct application value, pinpointing the contributions of individual flavonoids is challenging and is often confounded by interference from other substances.

In contrast, our approach adopts a holistic perspective and integrates the strengths of the aforementioned research methods. First, the current method utilizes a well-defined flavonoid mixture as a representative equivalent to reflect the overall characteristics of the flavonoid classes. This approach satisfies the research criteria of typical single-compound studies and is compatible with a cause–effect-based mechanistic research framework. Second, the approach provides comparative insights into the conserved and specific functions of flavonoid mixtures, particularly through comparisons to individual compounds. Due to mixture-based nature, this approach aligns closely with real-world dietary intake scenarios and extract-based research, where the concentrations of individual parent compounds are typically very low, but the overall flavonoid content remains high.^26^

The mixture strategy can be used to validate fragmented functional research, providing more representative evidence to support the health benefits, as observed in studies focusing on single compounds or extracts. Furthermore, this approach can serve as a reference framework in exploring novel functions or mechanisms of flavonoids, enabling the identification of flavonoid compounds with truly distinctive properties. However, it is crucial to recognize that, at both the class-wide and compound-specific levels, many aspects of flavonoid bioactivity are better viewed as a continuum rather than a binary classification. For instance, toxicity and certain downstream effects—such as activation of the ARE and NF-κB pathways—tend to be more generalized and cumulative, whereas direct interactions with molecular targets are often highly specific to particular compounds.

The class-wide effects observed in the mixture cannot be simply attributed to a linear combination of individual components. For instance, none of the single flavonoids exhibited toxicity at a concentration of 2.5 μM. However, when all the nontoxic flavonoids were pooled together, the resulting MIX_20_ displayed toxicity at 50 μM. Moreover, even when a high concentration of MIX_15_ was used to activate the luciferase reporter in a saturated manner, the system could still be further enhanced by the addition of single flavonoids. These observations underscore the nonlinear interactions within the mixture, indicating that the observed outcomes are influenced by the inherent complexity of the mixture and cannot be simply attributed to a few dominant contributing compounds.

Interestingly, as indicated by PI staining, the cell population may undergo a selection process at lower concentrations, whereby weaker or damaged cells are eliminated before a significant decline in overall viability occurs at higher concentrations. In parallel, under treatment with 20 μM MIX_20_, a decrease in cell numbers was observed, but no reduction in MTT values was detected at this concentration, indicating an increased MTT signal per cell. These observations are consistent with the hormesis hypothesis, which describes adaptive beneficial responses to low doses of otherwise toxic agents.^27^ This effect may be associated with mitochondrial stress, as MTT reduction partially occurs in mitochondria.

Recent studies have increasingly linked the bioactivities of flavonoids to their interactions with mitochondria.^28^ For instance, activation of mitoBKCa channels could explain transient MMP loss and certain cell-protective actions of flavonoids.^29^ In particular, a very low concentration of the mixture at 1 μM was sufficient to induce an MMP decrease, suggesting that individual components of the mixture may exert effects even at nanomolar concentrations.^30^ This finding is particularly relevant in the context of a daily diet, as it addresses concerns about the low bioavailability of dietary flavonoids.^31^ Given the complexity of the mixture’s effects, future studies could leverage proteomics, metabolomics, and other systems biology approaches to investigate potential mechanisms related to the induced mitochondrial perturbations and other cellular responses.

This study is intended as a proof of concept rather than a therapeutic application and thus does not address challenges related to ADME (absorption, distribution, metabolism, and excretion). In addition, the designation of our flavonoid mixture as a “class reference” was established in the current experimental models; accordingly, further validation in other experimental scenarios is needed. Meanwhile, it should be noted that some assay readouts are inevitably affected by pan-assay interference compounds (PAINS), of which flavonoids are a subset. Consequently, changes induced by flavonoids may not fully reflect the physiological benefits. Instead, PAINS-related effects may constitute part of the class-wide properties of flavonoids, which can also be captured by MIX_20_. Furthermore, the selection of 20 of the most representative flavonoids—out of over 10,000 known flavonoids—may have introduced some bias. For instance, the chosen flavonoid panel excluded anthocyanidins, which have extremely low bioavailability,^32^ and chalcone, which possesses a relatively different structure compared to other flavonoid subclasses. Future research could expand the panel to provide a more comprehensive understanding.

In conclusion, this proof-of-concept study shows that an equimolar cross-subclass cocktail of 20 well-studied flavonoids can serve as a pragmatic reference, highlighting class-wide bioactivities while diluting less conserved, compound-specific actions. In PC12 cells, the mixture reproduced the canonical flavonoid signature—activation of NF-κB, CRE, ARE, and NF-68 reporters, potentiation of NGF-driven neurite outgrowth, and a shared toxicity window—closely mirroring the effects of five benchmark flavonoids tested individually. At the same time, functions that are not broadly conserved, such as luteolin-induced mitochondrial depolarization or EGCG’s high-affinity binding to Aβ_1–42_, were diminished, illustrating an intrinsic buffering capacity within the blend. Although further validation across diverse cell types, *in vivo* models, and expanded flavonoid panels is required, the equimolar-mix strategy offers a scalable framework for elucidating class-level functions and mechanisms of phytonutrients. It also serves as a valuable tool for benchmarking individual molecules and assessing dietary or therapeutic formulations with complex repertoires.
